# T cell differentiation protein 2 facilitates cell proliferation by enhancing mTOR-mediated ribosome biogenesis in non-small cell lung cancer

**DOI:** 10.1007/s12672-022-00488-z

**Published:** 2022-04-18

**Authors:** Zhenying Lian, Xingyu Yan, Yutao Diao, Dayong Cui, Hongyan Liu

**Affiliations:** 1 Center for Basic Medical Research, Jinan Central Hospital, Shandong First Medical University, Jinan, 250013 Shandong China; 2 Institute of Basic Medicine, Shandong First Medical University, Jinan, 250062 Shandong China; 3 School of Life Sciences, Qilu Normal University, Jinan, 250200 Shandong China

**Keywords:** T cell differentiation protein 2, Cell proliferation, Ribosome biogenesis, mTOR targeted-therapy, Phosphoproteomics

## Abstract

Dysregulation of T cell differentiation protein 2 (MAL2) has been observed in multiple cancers, but its exact role in lung cancer is poorly understood. Here we report a role of MAL2 in accelerating cell proliferation in non-small cell lung cancer (NSCLC). MAL2 expression enhances cell proliferation in both cell and nude mouse models. Mechanistically, overexpression of MAL2 results in the hyper-activation of the MAPK/mTOR signaling pathway in NSCLC cells which leads to active ribosome biogenesis. Importantly, pharmacological inhibition of mTOR or MEK lowered the abundance of PCNA, a marker of tumor cell proliferation, and subsequently suppressed ribosome biogenesis, cell growth and xenograft growth in mouse model. MAL2 upregulation in clinical tumors is also linked to worse prognosis. Overall our data reveal that MAL2 is a potential diagnostic biomarker and targeting the MAL2/MAPK/mTOR signaling pathway may improve therapeutic strategy and efficacy for this subset of NSCLC patients.

**Supplementary Information:**

The online version contains supplementary material available at 10.1007/s12672-022-00488-z.

## Introduction

Human T cell differentiation protein 2 (MAL2), a member of the MAL protein family, was initially identified as a partner for tumor protein D52-like proteins in breast carcinoma [1]. The MAL2 protein has now been known to interact with the MHC-I complex and endosome-associated RAB proteins to inhibit the presentation of MHC-I molecules on cell membrane to drive immune evasion in breast cancer [2]. As an essential element of the machinery for basolateral-to-apical transcytosis both in hepatoma HepG2 cells [3, 4] and in human thyroid epithelial cells [5], MAL2 has been identified to boost the delivery of membrane-bound proteins and exogenous cargos from the basolateral to the apical surface. Several studies have identified MAL2 amplification and/or overexpression in breast cancer [6–9] and in other cancer types including primary ovarian carcinoma [10–12], pancreatic carcinoma [13, 14], prostate adenocarcinoma [15], oral squamous cell carcinoma [16] and head and neck squamous cell carcinoma [17]. A recent study revealed an oncogenic role of MAL2 in bladder carcinogenesis and its expression strongly associated with clinicopathological features of bladder cancer [18]. However, no study to date has examined whether MAL2 expression is increased in lung cancer, or its potential clinical significance.

The mechanistic/mammalian target of rapamycin (mTOR) is a conserved protein kinase that plays an essential role in cell growth and proliferation. As part of the mTOR complex 1 (mTORC1), mTOR regulates cell growth by promoting the biosynthesis of proteins, lipids and nucleic acids [19, 20]. It has emerged as a critical molecular in cancers because aberrant activation of mTORC1 signaling was observed in a large number of cancers. Importantly, mTORC1 activity was stimulated by the PI3K/Akt and Ras/MAPK pathways, and mutational events were frequently found in these pathways, such as mutations in Ras, Raf, PI3K and Akt oncogenes, and in the tumour suppressors, phosphatase and tensin homolog (PTEN) and TSC [21]. These mutations could cause constitutive activation of mTORC1 and stimulate anabolic processes driving tumor cell growth and proliferation. As mentioned, mTORC1 also promotes cell growth by driving tumor metabolism including ribosome biogenesis, which is a necessary larger program for protein synthesis of cells [22–24]. Several studies have demonstrated that using drugs to inhibit ribosome biogenesis might offer a viable therapeutic approach for cancer treatment [25–27]. Despite the success of strategies targeting the biogenesis or function of ribosome at the levels of rDNA gene transcription [25, 28, 29], ribosome protein (RP) synthesis [30], and mTORC1-dependent mRNA translation [31, 32] in cancers, the criteria for selection of patients who might benefit from inhibitors of ribosome biogenesis is largely unknown. Therefore, the identification and characterization of the tumor molecular subtype is still of great significance.

Our previous study has screened cancer-associated genes and identified *CCDC6-RET* and *TRA2B-DNAH5* fusion as novel oncogenic genes [33, 34]. Here, MAL2 was identified to be significantly upregulated in NSCLC tissues, and its high expression correlated with poor outcomes in lung cancer patients. The biological role of MAL2 in lung cancer cells was investigated both in vitro and in vivo. Overexpression of MAL2 resulted in the hyper-activation of the MAPK/mTOR signaling pathway in NSCLC cells, leading to active ribosome biogenesis which promoted cell proliferation and tumor growth. Importantly, pharmacological inhibition of mTOR or MEK lowered the abundance of PCNA, a marker of tumor cell proliferation, and subsequently suppressed cell growth and tumorigenesis in mouse model. Therefore, MAL2 is a potential diagnostic biomarker and targeting the MAPK/mTOR signaling pathway may improve therapy for this subset of NSCLC patients.

## Materials and methods

### Cell culture, animal models and clinical specimen collection

CRL-5872 cells were obtained from ATCC. A549, LC2-AD and NCI-H23 was purchased from cell bank of Shanghai institute of biochemistry and cell biology. Cells were cultured in DMEM (HyClone) or RPMI 1640 medium (GIBCO) with 8% or 10% FBS (BI), at 37 °C in 5% CO_2_ incubator. Four- to five-week-old, female Balb/C nude mice were purchased from Charles River Laboratories (Beijing) for experimentation. Regarding MAL2 alterations and the related patients’ survival in lung cancer, the information was downloaded from an open access database which is available at http://www.cbioportal.org. Altogether, we analyzed 522 cases of adenocarcinomas (TCGA, Firehose Legacy) and 178 cases of squamous cell carcinomas (TCGA, Nature 2012), and of these adenocarcinomas, 512 cases contain overall survival time.

### Plasmid construction and lentivirus infection

*MAL2* was cloned into pCDH-CMV-EF1-CoGFP (System Biosciences). *MAL2* with C-terminally fused 3× Flag tag was cloned into GV492 which was performed by Shanghai Genechem Co., LTD. The shRNAs toward human *MAL2* were cloned into pLKO.1 vector (Addgene). The target sequences of shRNAs were as follows:

shMAL2-1: ccggccTGCATGATTTGCATTGCAActcgagTTGCAATGCAAATCATGCAggtttttg; shMAL2-2: ccggctAACTGGAACTTCCTGGATTctcgagAATCCAGGAAGTTCCAGTTagtttttg. Lentiviral package and infection was performed as follows: viral particles were produced in HEK-293T cells co-transfected with pCDH (or pLKO) constructs and packaging plasmids pCMV-VSVG/delta8.2 (System Biosciences) in DMEM media. The progeny viruses released from HEK-293T cells were filtered, collected and used to infect cells.

### Real-time PCR

Real-time PCR was performed on BIO-RAD CFX96 Real-Time System and the results were calculated by the comparative cycling threshold (Ct) quantization method. The relative expression levels were analyzed using 2^−∆∆Ct^ method and normalized to the levels of *GAPDH*. SYBR-green Master Mix (Roche) was used to detect and quantify the expression of the target gene. The following primers were used: *GAPDH*, 5′-GCGACACCCACTCCTCCACCTTT-3′ (forward) and 5′-TGCTGTAGCCAAATTCGTTGTCATA-3′ (reverse); *MAL2*, 5′-CGACATCCTGCGGACCTACT-3′ (forward) and 5′-TGGCTGCTGCTTCCAATAAA-3′ (reverse); *5′ ETS* (forward) 5′-CAGGTGTTTCCTCGTACCG-3′ and 5′-GCTACCATAACGGAGGCAGA-3′ (reverse); *45S* pre-rRNA, 5′-GCCTTCTCTAGCGATCTGAGAG-3′ (forward) and 5′-CCATAACGGAGGCAGAGACA-3′ (reverse). *TIF 1A*, 5′-GCCTCCTGCCATGTACAGTT-3′ (forward) and 5′-CAAAAATGCTTCTGCAAATCC-3′ (reverse); *NCL*, 5′-CATGGTGAAGCTCGCAAAG-3′ (forward) and 5′-TCACTATCCTCTTCCACCTCCTT-3′ (reverse).

### Western Blotting

Lung cancer cells or tumor tissues were lysed and sonicated in RIPA buffer supplemented with protease inhibitors (Solarbio, Beijing). Protein extracts were boiled in sample buffer (Solarbio), separated by SDS-PAGE and transferred to nitrocellulose filter membranes (Millipore). After blocking in PBS/Tween-20 containing 5% BSA, the membranes were incubated with the primary antibodies. The following antibodies were used: PCNA (#2586), GAPDH (#5174), p-Erk1/2 (#9101), Erk1/2 (#9102), p-AKT (S473, #9271), AKT (#2920), p-RPS6 (#2215), RPS6 (#2217), p-mTOR (#5536) and mTOR (#2983), purchased from Cell Signaling Technologies; MAL2 from Bioss (bs-7175R). β-Actin (ab179467) was purchased from abcam. Signal visualization was detected with ECL substrate (millipore) and a Biotek imaging system. Immunohistochemistry was performed as previously described [35].

### Gene functional assays

To measure cell proliferation ability, cells were seeded in 96-well plates at a density of 3 × 10^3^ per well, and cell growth rate was assessed with the 3-(4,5-dimethylthiazol-2-yl)-2,5-diphenyltetrazolium bromide (MTT) kit (Roche Diagnostics). The MTT assays in each cell line repeated three times, respectively. Cell proliferation ability was further confirmed by soft agar colony formation assay; 8 × 10^3^ cells were seeded in 6-well plates, and after 3 weeks of culture cell colonies were counted by crystal violet staining. To detect the effects of inhibitors (selumetinib, rapamycin, torin; Selleck) on cell growth we performed colony-formation assay. After incubation for 3 days with indicated inhibitors, the cells in 12-well plates were fixed with 4% paraformaldehyde (PFA) and stained with 0.1% crystal violet solution to visualize their colony-forming ability.

### EU and EdU staining

For both 5-ethynyl uridine (EU, invitrogen) staining and 5-ethynyl-2′-deoxyuridine (EdU, Beyotime, Shanghai) staining cells were pulsed with azide-Click-IT technology as manufacturer’s protocol. Cells were visualized using MD ImageXpress Micro (Molecular Devices) High-Content Screening microscope. Each experiment was repeated at least three times. For EU assays, fluorescent signal intensity was quantified using ImageJ software. Images were converted to 8-bit depth; signal intensity was quantified by measurement of ‘Integrated Density’. The ‘Integrated Density’ value was then divided by the number of cells in the same field. For EdU assays, the number of EdU positive cells was counted, and this number was then divided by the total number of cells in the same field, finally the data were converted to a percentage value.

### Quantitative phosphoproteomic analysis

Lysates of NCI-H23 cells infected with control lentivirus or overexpressing MAL2 were further lysed with sonication at 4 °C for 3 min (80 W, on 1 s and off 1 s) and centrifuged at 12,000×*g* at 4 °C for 10 min to remove insoluble particles, then centrifuged one more time and the supernatant was collected. The samples were fractionated using sequencing-grade trypsin in 100 mM TEAB buffer (Sigma). Then, the samples were labeled using the TMT label reagent (Thermo scientific) according to the manufacturer’s instructions. Phosphorylated peptides were enriched by using titanium dioxide beads (TiO_2_). Mass spectrometry analysis was performed by Shanghai OE-biotech (China) with an EASY-nLCTM 1200 system (Thermo, USA) in Q-Exactive mass spectrometer equipped with a Nanospray Flex source (Thermo, USA). The data were processed with Proteome Discoverer™ 2.2 (Thermo, USA) software against the Homo sapiens Uniprot database. The phosphorylatedproteins with an average fold change (FC > 1.2; *P* < 0.05) in the experimentally treated groups were considered to be the differentially accumulated proteins.

### A549 xenograft tumorigenesis assay

5 × 10^6^ A549 control cells or A549-MAL2 cells in 100 µl PBS were injected into the dorsal flank of 5 week-old randomly grouped (6 mice per group) female BALB/c nude mice. For drug therapy, nude mice with A549-MAL2 tumors were randomly assigned to either vehicle or rapamycin treatment groups. Rapamycin and its solvent control were given intraperitoneally at a dose of 2 mg/kg every 2 days beginning at 9 weeks to an age of 11 weeks. Tumor growth was monitored regularly for up to 19 days by measuring tumor diameters with digital calipers. Tumor volume was calculated by the formula: volume = 0.52 × length × (width)^2^.

### Statistical analysis

The statistical significance between the two groups was analyzed by Student’s *t*-test using Graphpad Prism 7.0 (GraphPad Software, Inc.). The variance among multiple groups was analyzed by oneway analysis of variance (ANOVA). Overall survival (OS) curves were produced by the Kaplan–Meier method. Error bars represent SEM. *P* < 0.05 was considered to be significant.

## Results

### MAL2 is up-regulated in non-small cell lung cancer

**Fig. 1:**
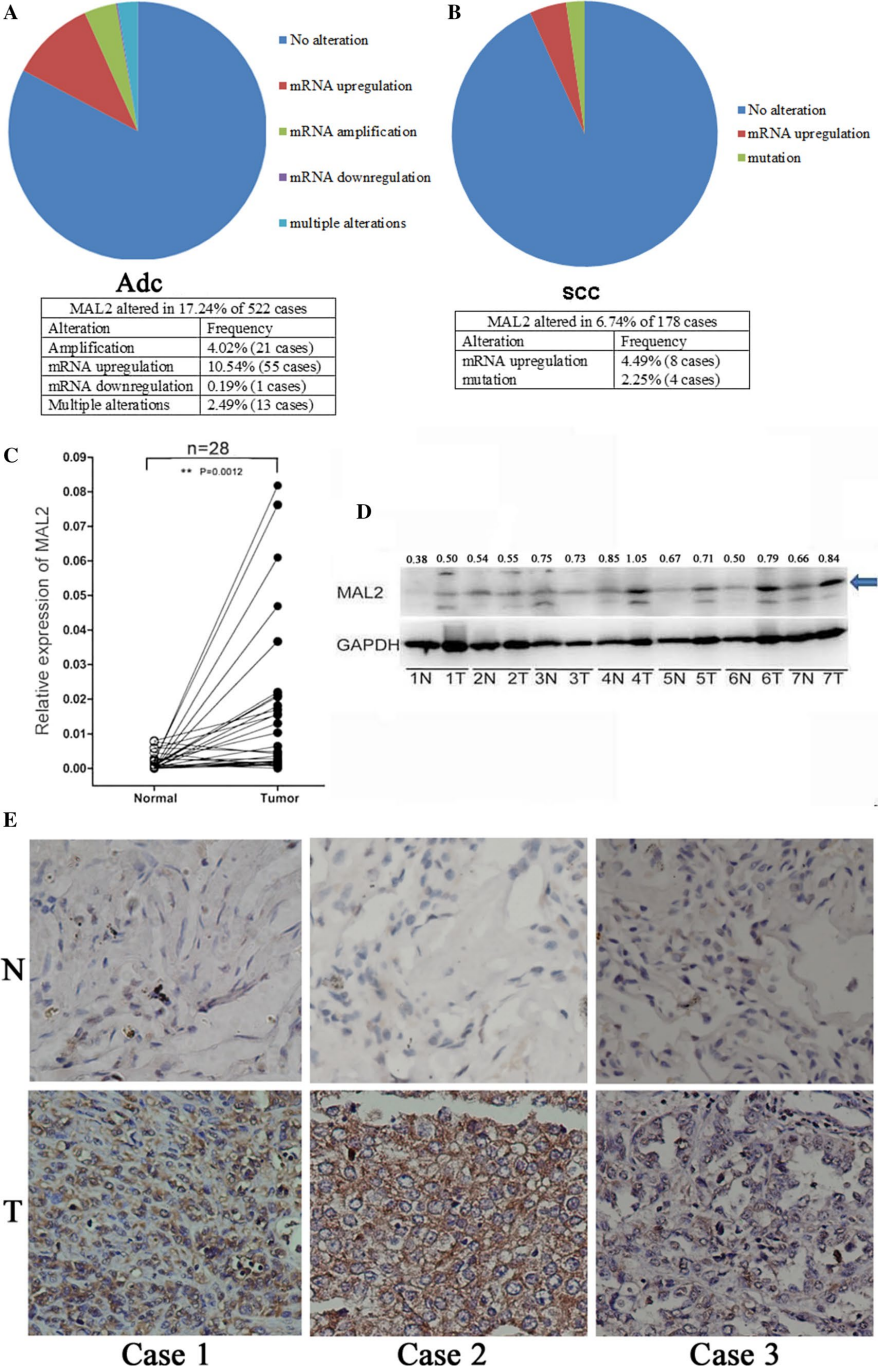
The relative mRNA expression and protein expression of MAL2 in tumor and paired peritumoral tissues. **A**, **B** The expression of MAL2 was altered in lung adenocarcinomas and lung squamous cell carcinomas, based on the analysis of 522 TCGA adenocarcinoma samples and 178 TCGA lung squamous cell carcinomas. **C** MAL2 mRNA levels were quantified in 28 pairs of NSCLC tissues and adjacent normal tissues using quantitative real-time PCR. The statistical significance was analyzed by oneway analysis of variance. **D**, **E** MAL2 protein levels were assayed by western blot and immunohistochemical staining. N: non-cancerous tissue; T: tumor. Numbers above the lanes represent the ratio of MAL2 expression levels normalized to GAPDH expression levels by measuring optical density value

To study how prevalent is MAL2 in tumor samples, we analyzed transcriptomics data to compute *MAL2* transcription levels in tumor specimens from the cancer genome atlas (TCGA). We found that both adenocarcinoma (Adc) and lung squamous cell carcinoma (SCC) have aberrant expression of *MAL2*. It was upregulated in 10.54% of Adc cases and in 4.49% of SCC cases (Fig. 1A, B). The real-time PCR validated the higher expression of *MAL2* in lung tumor tissues compared with paired noncancerous tissues (Fig. 1C). Moreover, the expression levels of MAL2 protein in NSCLC tissues were frequently higher than that in the paired noncancerous tissues (Fig. 1D). Immunohistochemistry analysis was conducted to further validate the expression level of MAL2 in NSCLC tissues and paired peritumoral tissues. As shown in Fig. 1E, MAL2 signal was obviously stronger in lung cancer tissues compared with peritumoral tissues.

### High expression of MAL2 facilitates the proliferation of lung cancer cells in vitro and in vivo

**Fig. 2:**
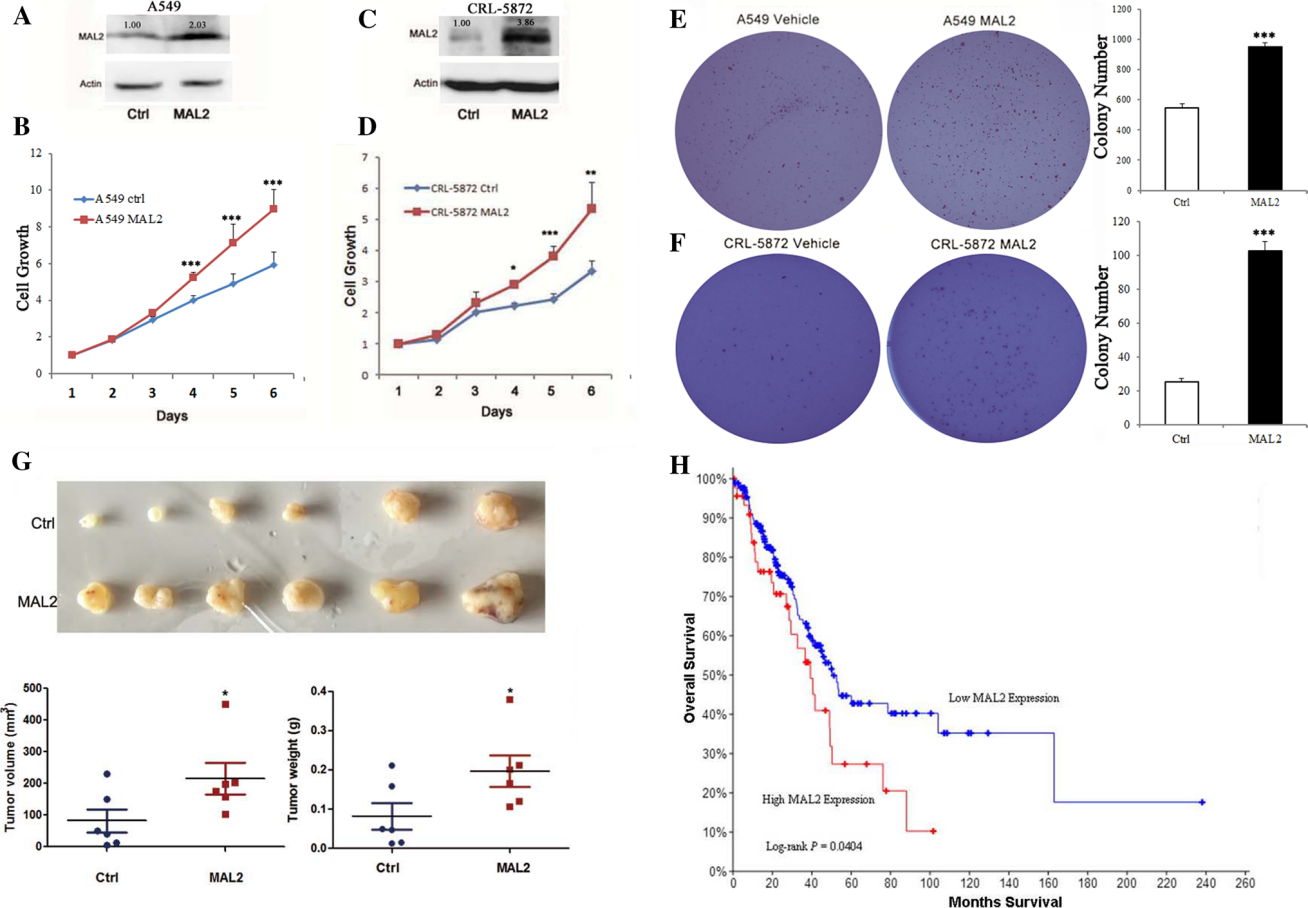
MAL2 promotes NSCLC progression. **A**, **C** Western Blot analysis of MAL2 expression in lung cancer cell lines with or without MAL2 overexpression. Actin was used as loading control. **B**, **D** A549 and CRL-5872 expressing vector control or over-expressing MAL2 were assayed for cell proliferation. The values represent the average ± SEM determined from at least three independent experiments. **E**, **F** MAL2 promoted anchorage-independent growth of A549 and CRL-5872 cells in soft agar assay. Three independent experiments were performed. **G** MAL2 promoted tumor growth in A549 xenograft assay. Nude mice were injected with lentivirus-GFP or lentivirus-MAL2 A549 cells and photos of xenograft tumors, tumor volume and tumor weight were shown. N = 6 mice for each group. **H** Kaplan–Meier curve of overall survival of non-small cell lung cancer patients analyzed for MAL2 expression in the 512-patient-TCGA cohort. The blue line shows the overall survival of patients with low MAL2 expression, the red line shows patients with high expression of MAL2

To study the effect of MAL2 on cell proliferation, MTT and colony formation assay were performed. Compared to the control A549 cells, cells expressing MAL2 showed elevated cell growth (Fig. 2A, B; Figure S1). We extended this analysis to two additional lung adenocarcinoma cell lines, NCI-H23 and CRL-5872, and found that MAL2 overexpression resulted in an increased cell proliferation similar to observed in A549 MAL2 expressing cells (Figures S2, S3 and Fig. 2C, D). Moreover, overexpression of MAL2 significantly enhanced the colony formation abilities of A549 and CRL5872 cells (Fig. 2E, F). Next, we confirmed the function of MAL2 in cell proliferation by knockdown of MAL2 in CRL-5872 and LC2-AD cells, two human LUAD cell lines with high endogenous expression of MAL2, and found that MAL2 knockdown significantly decreased cell growth (Figure S4). To determine whether the promotion of cell proliferation manifests in vivo, we generated both control and MAL2-overexpressed A549 xenografts in nude mice. MAL2 overexpression markedly promoted tumor growth as indicated by increased tumor volume and weight (Fig. 2G). In addition, the high expression of MAL2 was associated with short overall survival time (Fig. 2H). Collectively, these results demonstrate that MAL2 upregulation promotes NSCLC progression.

### mTORC1 pathway is the downstream signaling of MAL2

**Fig. 3:**
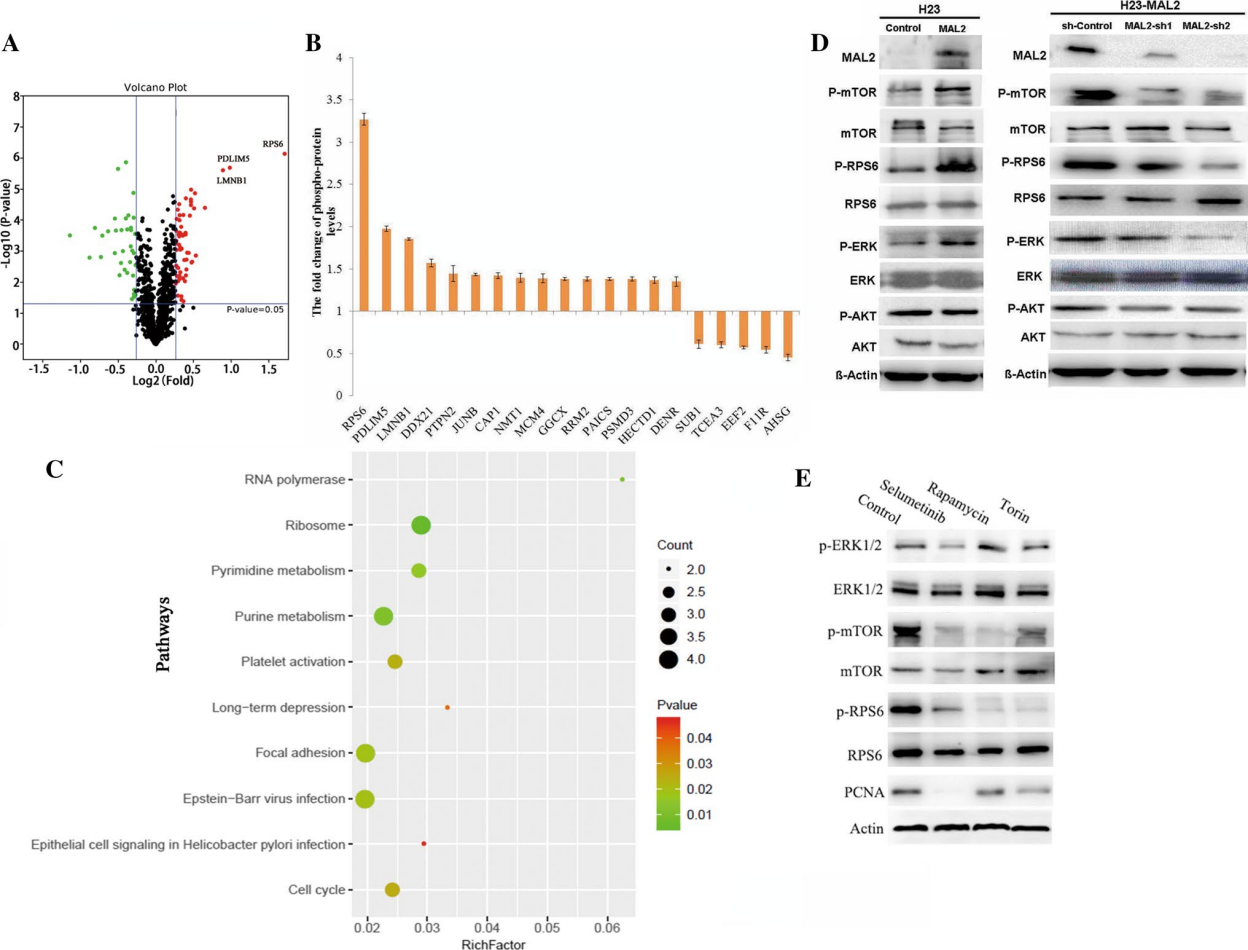
MAL2 regulates the MAPK/mTOR signaling pathway in NSCLC. **A** Differentially phosphorylated proteins between control cells and MAL2 overexpression NCI-H23 cells. Red and green colors, upregulated and downregulated phosphoproteins in MAL2 overexpression cells, respectively (FDR < 0.01 from Wilcoxon signed-rank test and fold change > 1.2). **B** Fold changes of top 15 upregulated proteins and top 5 downregulated proteins identified from the quantitative phosphoproteomic analysis. **C** Functional annotation clustering of proteins regulated by MAL2 in NCI-H23 cells. Enriched groups listed by their gene ontology term are ranked on the basis of the significant enrichment scores. **D** Left panel: immunoblotting analysis for mTOR, RPS6, AKT, and ERK phosphorylation in NCI-H23 cells with or without overexpression of MAL2. Right panel: immunoblotting analysis of mTOR, RPS6, and ERK phosphorylation after MAL2 silencing in NCI-H23 cells stably overexpressing MAL2. **E** MAL2 activates mTOR through modulating ERK1/2. Western blot analysis of p-ERK1/2, total ERK1/2, p-mTOR, total mTOR, p-RPS6, total RPS6, and PCNA in A549-MAL2 cells. MEK inhibitor selumetinib (10 µmol/l), mTOR inhibitors rapamycin (100 nmol/l) and torin (250 nmol/l) incubated cells for 48 h

To begin investigating the mechanism by which MAL2 influences cell proliferation and tumor growth, we performed phosphorylated proteomics to measure the changes in protein expression of NCI-H23 with or without MAL2 overexpression. Isobaric tandem mass tags (TMT)-based phosphoproteomics identified 1128 highly reliable phosphoproteins, and a total of 95 phosphoproteins quantified across all six samples and 349 phosphosites were included in subsequent analyses (Table S1). Analysis for differential phosphorylated protein expression revealed 59 up- and 36 down-regulated proteins (p < 0.05, > 1.2-fold change) in cells constitutively expressing *MAL2* (Fig. 3A, Table S1). Gene Ontology (GO) enrichment analysis revealed that cellular component organization or biogenesis proteins, including gamma-glutamyl carboxylase (GGCX), ribonucleotide reductase regulatory subunit M2 (RRM2), phosphoribosylaminoimidazole carboxylase and phosphoribosylaminoimidazole succinocarboxamide synthetase (PAICS), were up-regulated in MAL2-overexpressing cells (Fig. 3B). GO enrichment analysis also revealed significant enrichment in mTORC1 signaling, among which phosphorylated ribosome protein S6 (RPS6) was increased > threefold in MAL2-overexpressing cells (Fig. 3B). RPS6 is a structural component of the 40S ribosomal subunit. Functional annotation clustering via KEGG revealed “ribosome biogenesis” (p < 0.01) as the top scoring functional group (Fig. 3C). Closer analysis of the up-regulated protein set (> 1.2-fold change) indicated significant enrichment for “RNA polymerase” (p < 0.05), “purine metabolism” (p < 0.05), “pyrimidine metabolism” (p < 0.05) and “focal adhesion” (p < 0.05) among the top functional groups (Fig. 3C). Immunoblotting analysis of NCI-H23 protein lysates confirmed increased phosphorylation of RPS6, mTOR and ERK in cells expressing *MAL2* versus vector control. To provide additional evidence to support the regulation of these candidates by MAL2, we used NCI-H23-MAL2 cells to infect with lentivirus expressing shRNA against MAL2 (shMAL2), or its negative control (sh-control). Western blotting analysis confirmed that MAL2 expression was significantly down-regulated in cells infected with shMAL2 with respect to the negative control cells. The results also demonstrated that MAL2 knockdown can decrease the phosphorylation levels of RPS6, mTOR and ERK (Fig. 3D).Taken together, the findings indicate that MAL2 could activate mTORC1 signaling in NSCLC.

### The activation of mTORC1 by activated MAPK signaling

As PI3/AKT and MAPK are the main activator of the mTOR pathway, we investigated whether these upstream molecules of the mTOR pathway were also modulated by MAL2. As our data shown MAL2 knockdown could decrease the phosphorylation levels of ERK, while did not affect the phosphorylation level of AKT (Fig. 3D). We further examine the effect of MEK/ERK on mTOR signaling in A549-MAL2 cells upon inhibitor treatments (selumetinib, MEK1/2; rapamycin, pan-mTOR; Torin, mTORC1) (Fig. 3E). Selumetinib treatment inhibited the phosphorylation of mTOR, RPS6, and ERK. Both rapamycin and torin treatment significantly decreased the phosphorylation of mTOR and RPS6, while they did not decrease the phosphorylation of ERK suggesting that MEK/ERK is the upstream signaling of mTOR. As the proliferation marker, proliferating cell nuclear antigen (PCNA) was also confirmed to being markedly reduced by both selumetinib and mTOR inhibitor. Together, these data suggest that MAL2 expression drives deregulation of MEK/ERK/mTOR pathway and it might contribute to the effect of MAL2 on cell proliferation in NSCLC.

### MAL2 facilitates ribosome biogenesis

**Fig. 4:**
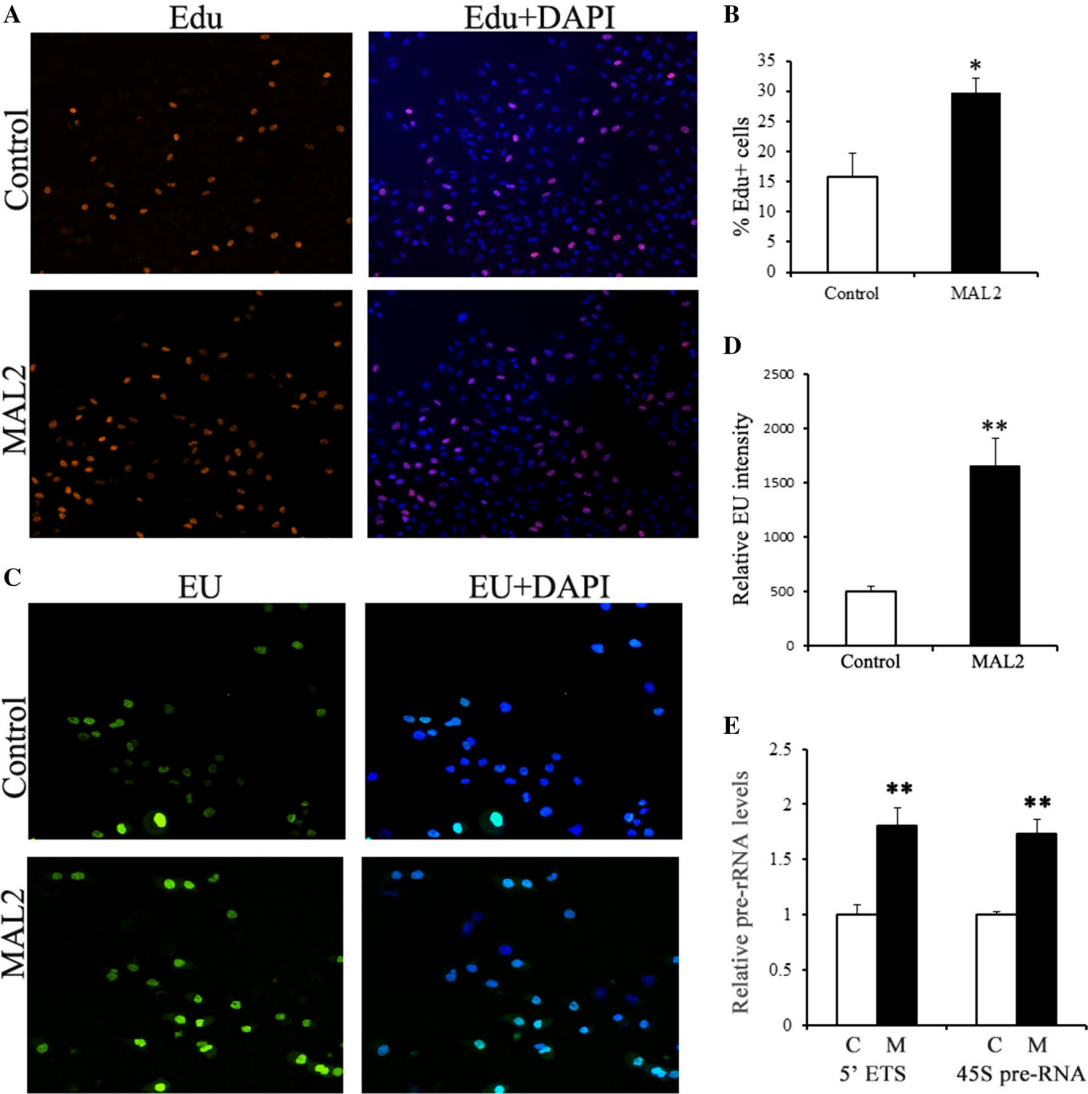
Enhanced rRNA synthesis and cell proliferation in A549 cells with MAL2 over-expression. **A**, **C** DNA synthesis (EdU, red) and rRNA synthesis (EU, green) in control cells and MAL2 over-expression cells. Three independent experiments were performed and six replicate wells for each experiment. **B**, **D** Quantifications of EdU and EU in **A**, **C**, *p < 0.05, **p < 0.01. **E** qRT-PCR of 5′ ETS and 45S (pre)-rRNA transcript in control cells and MAL2 over-expression cells. Data in **E** are mean ± SEM of three replicates; t-test, **p < 0.01

Ribosome biogenesis is a canonical hallmark of cell growth and proliferation. Because MAL2 could promote cell proliferation and was coincident with increasing levels in RNA polymerase and ribosome proteins, we examined MAL2’s potent effects on ribosome biogenesis. To visualize nascent rRNA and DNA synthesis, MAL2-overexpressing cells were pulsed with ethynyluridine (EU; rRNA synthesis) [22] and 5-ethynyl-2′-deoxyuridine (EdU; DNA synthesis). These experiments revealed that EU levels were > twofold higher in the MAL2-overexpressing cell population and highly localized to the nucleolus (Fig. 4C, D). In mammals, RNA polymerase I (Pol I) transcribe 47S pre-rRNA to generate the mature rRNA species 5.8S, 18S and 28S. The 47S pre-rRNA contains the external transcribed spacer (5′ETS) of rDNA genes. We observed that 5′ETS levels were significantly increased in MAL2-overexpressing cells. Overexpression of MAL2 elevated ribosome biogenesis was further confirmed by the induction of *pre-45S* rRNA transcripts in these cells (Fig. 4E). MAL2-overexpressing cells also exhibited a concomitant increase in the number of EdU^+^ cells (Fig. 4A, B). We conclude from these findings that the level of ribosome biogenesis was induced by MAL2 and was concomitant with an increase in cell proliferation.

### The impacts of mTORC1 and MEK inhibitors on ribosome biogenesis

**Fig. 5:**
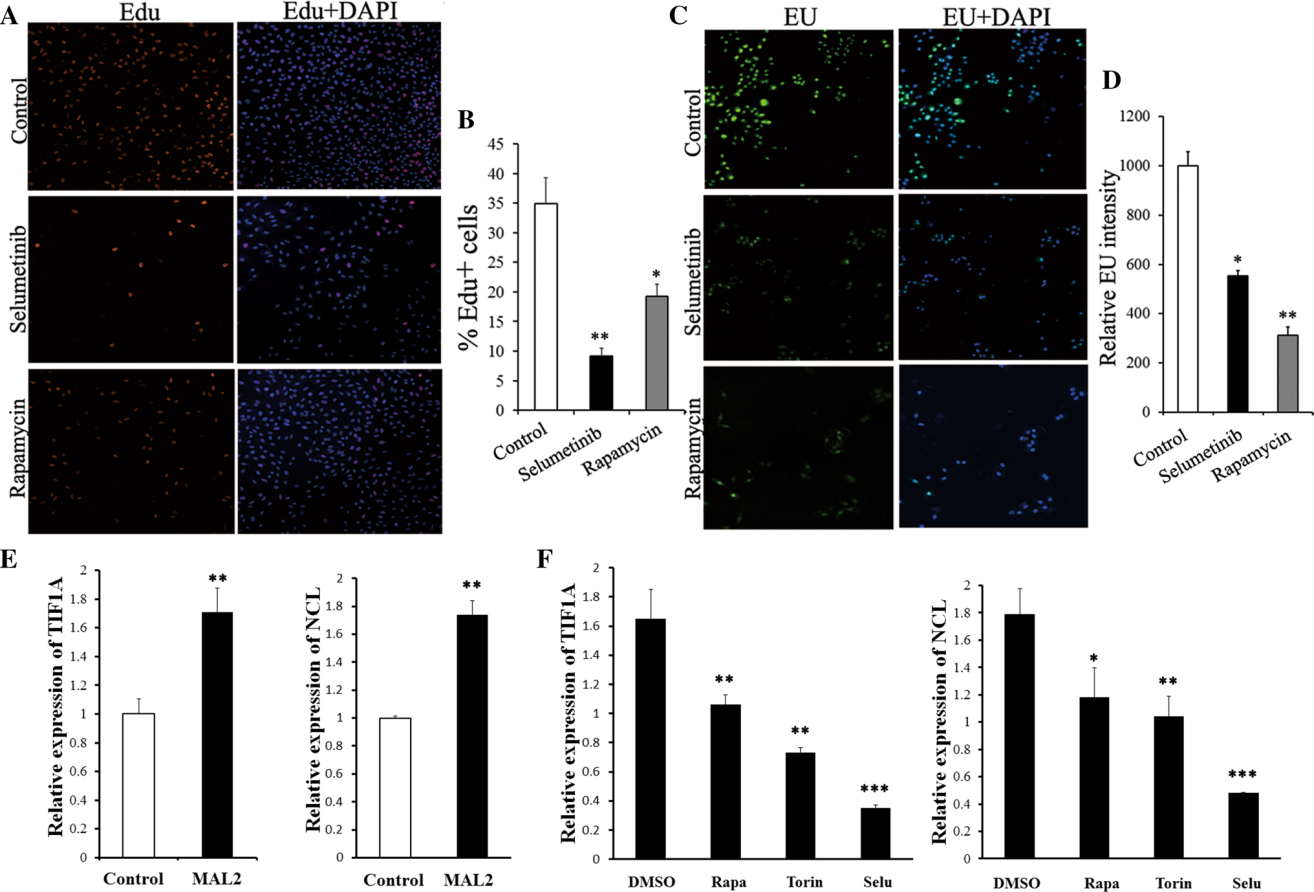
Ribosome biogenesis inhibition correlates with reduced cell proliferation. **A**, **C** A549 cells with MAL2 overexpression treated with or without 10 µmol/l selumetinib or 100 nmol/l rapamycin for 48 h before analysis of DNA synthesis (EdU, red) and rRNA synthesis (EU, green). **B**, **D** EdU and EU quantifications from **A** and **C**. Data are mean ± SEM of three replicates; t-test, *p < 0.05, **p < 0.01. **E** MAL2 overexpression induced TIF1A and NCL transcription. qRT-PCR showing increase of TIF1A and NCL expression in A549 cells with overexpression of MAL2. Data are presented as mean ± SEM of three replicates; t-test, **p < 0.01. **F** The mTOR- and MEK-specific inhibitors reduced TIF1A and NCL transcription. A549 cells with overexpression of MAL2 were treated for 48 h with rapamycin, torin and selumetinib. The TIF1A and NCL transcription were detected by qRT-PCR. Data are presented as mean ± SEM, n = 3. *p < 0.05, **p < 0.01, ***p < 0.001

One convinced function of mTORC1 is to promote ribosome biogenesis as a key part of a larger program to increase the protein synthesis capacity of cells [22–24]. Studies have shown the functions of mTORC1 in inducing translation of mRNAs which encode the ribosomal protein subunits, and in rRNA synthesis. To evaluate whether the inhibition of MAPK-mTOR pathway led to disruption of ribosome biogenesis, we treated MAL2 over-expressed A549 cells in culture with MAPK-mTOR inhibitors (Fig. 5). The administration of rapamycin significantly reduced EU incorporation. Consistently, the MEK inhibitor, selumetinib, also reduced rRNA synthesis (Fig. 5C, D). Hence, this finding further indicated that inhibition of the upstream factors of mTORC1 could decrease ribosome biosynthesis. The mTORC1 synergistically regulates three essential RNA Polymerase I (Pol I)-specific transcription factors, UBF, SL-1, and TIF1A, and relieves MAF1-mediated inhibition of RNA Polymerase III (Pol III) transcription to stimulate rRNA synthesis [24, 36, 37]. Cells with overexpressing MAL2 displayed increased TIF1A, an essential component of the Pol I complex for initiation of rRNA syhthesis and Nucleolin (NCL) expression, another eukaryotic nucleolar phosphoprotein, which is involved in the synthesis and maturation of ribosomes (Fig. 5E). Furthermore, the expression of TIF1A and NCL was significantly downregulated by rapamycin, torin, as well as selumetinib in A549 cells overexpressing MAL2 (Fig. 5F). Collectively, these results demonstrate that MAPK-mTOR inhibitors could disrupt the transcription of key rRNA synthesis transcripts, thereby reduced ribosome biogenesis.

### The impacts of mTORC1 and MEK inhibitors on cell proliferation

**Fig. 6:**
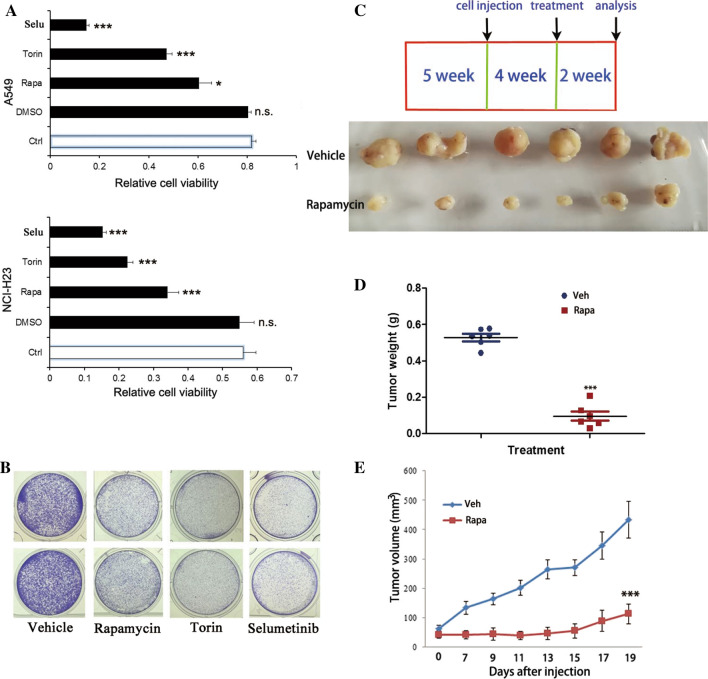
Targeting mTOR using mTOR- or MEK-specific inhibitors efficiently inhibits the growth of lung cancer cells with MAL2 overexpression. **A** Selumetinib (10 µmol/l), torin (250 nmol/l) and rapamycin (100 nmol/l) significantly inhibited cell proliferation of A549-MAL2 and H23-MAL2 cells upon 48 h treatment. **B** Rapamycin (100 nmol/l), torin (250 nmol/l) and selumetinib (10 µmol/l) reduced cell growth. Colony formation of A549-MAL2 cells were assayed by crystal violet staining after 72 h treatment. **C**–**E** Nude mice were injected with lentivirus-MAL2 A549 cells and treated with 2 mg/kg rapamycin or vehicle control once every 2 days since tumor establishment for 2 weeks (**C**). Tumor growth was monitored every 2 days after intraperitoneal injection (**E**), and tumor weight was measured after autopsy (**D**). N = 6 mice for each group

To determine whether *MAL2*-mediated cell growth is dependent on the mTOR pathway, we detected cell proliferation and colony formation after treatment of *MAL2*-expressing cells with mTORC1 inhibitors rapamycin or torin. After inhibition of mTOR, the proliferation and colony formation of A549-MAL2 (Figs. 5A, B and 6A, B) and the proliferation of NCI-H23-MAL2 cells (Fig. 6A) were significantly decreased. Similarly, the proliferation and colony formation of A549-MAL2 cells were also inhibited by MEK inhibitor (Figs. 5A, B and 6A, B). Next, we investigated whether the control cells are affected by these inhibitors. The proliferation and colony formation assays showed that, upon rapamycin, torin or selumetinib treatment, significant decreases in cell growth were observed for A549-empty vector cells (Figure S5). The proliferation of NCI-H23-empty vector cells was also inhibited by rapamycin, torin and selumetinib (Figure S5A). These results indicate that the mTORC1 and MEK inhibitors have an impact on the control cells. However, the inhibition efficiency was much higher in MAL2 overexpression cells than that in the control cells (Fig. 6A, B and Figure S5). These results indicate that the dysregulated MAL2 expression resulted in over-activation of MAPK and mTORC1 pathways which are required by MAL2 to fully elicit its pro-proliferative phenotype.

### Inhibition of mTORC1 signaling prevents tumorigenesis in a xenograft model

To further investigate the therapeutic effects of rapamycin treatment in human lung adenocarcinoma, we treated mice bearing lung tumors with rapamycin. We injected A549 cells that overexpressed MAL2 into nude mice. After tumor establishment, the mice were treated every 2 days with either 2 mg/kg mTOR inhibitor rapamycin or vehicle. As shown in Fig. 6C, tumor size was significantly decreased in the treatment groups compared with the control group. Rapamycin treatment was able to decrease the growth rate, tumor volume and tumor weight (Fig. 6D, E). Moreover, rapamycin treatment decreased cell density, PCNA expression, mTOR and RPS6 phosphorylation (Figs. 3 and 6B). These data demonstrate that MAL2 promotes tumor formation of human lung adenocarcinoma cells, while mTOR inhibitors suppress tumor growth in established tumors using human lung cancer cell xenografts.

## Discussion

Many novel drive mutations have been investigated in NSCLC, including FGFR pathway aberrations, MET Ex14 point mutation and several fusion genes. The discovery of these drive mutations not only significantly improves clinical outcomes but also facilitates the development of new therapy strategy. Here, we observed that MAL2 was overexpressed in NSCLC and promoted lung cancer growth. Importantly, we have demonstrated that MAL2 enhanced ribosome biogenesis and cell proliferation via activation of MAPK/ERK/mTORC1 signaling pathway. mTOR functions as a key regulatory protein in normal cell growth, survival, metabolism and development. Dysregulation of mTOR signaling has been frequently observed in wide variety of cancers, including lung cancer. Targeting of mTOR is attractive, indeed, some of the mTOR inhibitors have been development to treat cancer, including rapamycin analogs (rapalogs), everolimus and temsirolimus [38]. It is necessary to evaluate if there are predictive biomarkers that may guide the stratification of patients in clinical trials who most likely benefit from treatment with mTOR inhibitors. The aberrant expression of MAL2 leads to MAPK/mTOR activation which may predict sensitivity to MEK or mTOR inhibition. Thus, patients with MAL2 overexpression might be benefit from an existing treatment strategy.

Significant upregulation of key enzymes of the genome replication process (RRM2, ribonucleotide reductase regulatory subunit M2; MCM4, mini-chromosome maintenance complex component 4, etc.) was observed in MAL2 expressing cells, indicating enhanced demands for biological metabolism in NSCLC. mTORC1 promotes cell growth primarily through the activation of key anabolic processes, and enhancing ribosome biogenesis is a major function of mTORC1 [22]. It is thus not surprising that ribosome proteins (RPS6, ribosomal protein S6, a structural component of the 40S ribosome subunit; RPS27, ribosomal protein S27) are hyperactivated after mTOR activation. rRNA is a major component of the ribosome and, as such, carcinogenesis requires an increase in its synthesis [39, 40]. In addition to stimulating the activity of ribosomal proteins, mTORC1 also positively regulates the Pol I- and Pol III-dependent transcription of the different classes of ribosomal RNAs [41]. We observed that MAL2 induced RNA polymerases expression (POLR3E: RNA polymerase III subunit C5; TWISTNB: DNA directed RNA polymerase I subunit RPA43). Two enzymes, DDX21 (DExD-box helicase 21, an RNA helicase) and PAICS (phosphoribosylaminoimidazole carboxylase and phosphoribosylaminoimidazole succinocarboxamide synthetase), known to function in ribosomal RNA biogenesis, pyrimidine and purine biosynthesis, were also found to be enhanced in response to mTORC1 activation induced by MAL2. Studies have demonstrated that mTORC1 signaling promotes de novo pyrimidine and purine synthesis which is required for cell growth [42–44].Treatment of cells with mTOR- or MEK-specific inhibitors resulted in reduced ribosome synthesis and cell growth, confirming the important role that the MAL2/MAPK/ERK/mTORC1 pathway plays in this anabolic process as well as in cell proliferation.

As a tetraspanning membrane protein MAL2 has been implicated in regulating transcytosis [4], and the transcytotic efflux from early endosomes to the subapical compartment in polarized, hepatic WIF-B cells required cholesterol and glycosphingolipids [45], in other words, MAL2 plays a lipid-dependent transport function. In addition to its role in transcytosis, MAL2 also regulates pIgA-R delivery from the Golgi to the plasma membrane [46]. It is well-known that mTORC1 senses and responds to fluctuations in the levels of intra- and extracellular nutrients to modulate cellular growth, metabolism and survival. Thus, it is very likely that the traffic of MAL2 and the transported substance in the cell surface activate mTOR signaling. When active, mTORC1 promotes anabolic processes such as protein, lipid and nucleotide synthesis through phosphorylation of its downstream effectors thus inducing ribosome biogenesis and cell growth. Further studies are needed to understand the mechanistic details of MAL2-mediated augmentation of ribosome synthesis in lung cancer.

## Conclusions

In summary, MAL2 overexpression enhances cell proliferation and ribosome biogenesis via hyper-activation of the MAPK/mTOR signaling pathway in NSCLC. MAL2 may be used as a novel predictive biomarker to guide the stratification of patients in clinical trials who most likely benefit from treatment with mTOR inhibitors.

## Supplementary Information

Below is the link to the electronic supplementary material.

Supplementary file 1. Figure S1. Cell lysates from A549 cells expressing vector control or over-expressing MAL2 were immunoblotted for human MAL2. Molecular weight standards are indicated on the right in kDa. The predicted molecular weight of MAL2 is 19 kDa. The band above 35 kDa was also detected which might be the glycosylated MAL2. A diffuse set of bands were also shown that has been previously described by others.

Supplementary file 2. Figure S2. NCI-H23 cells expressing vector control or over-expressing MAL2 were assayed for cell proliferation. Data are mean ± SEM of three replicates from a representative experiment of two independent experiments; t-test, *p < 0.05.

Supplementary file 3. Figure S3. Cell lysates from CRL-5872 cells expressing vector control or over-expressing MAL2 were immunoblotted for human MAL2. The size of 19 kDa MAL2 was shown in Fig. 2C.

Supplementary file 4. Figure S4. Knockdown effects of MAL2 in CRL-5872 and LC2-AD cell lines. (A, C) Western blotting showed the reduced MAL2 protein levels after 72 h infection of lentivirus with shMAL2 compared with lentivirus with scrambled control shRNA. (B, D) Relative growth curve of CRL-5872 and LC2-AD cells with or without MAL2 knockdown. CRL-5872 cells were seeded in 96-well plates at a density of 3 × 10^3^ per well and LC2-AD cells were seeded in 96-well plates at a density of 4 × 10^3^ per well **P < 0.01, ***P < 0.001.

Supplementary file 5. Figure S5. The effects of mTOR- or MEK-specific inhibitors on cell growth. (A) A549 and NCI-H23 with empty vector cells (A549 EV, NCI-H23 EV) were treated for 48 h with rapamycin (100 nmol/l), torin (250 nmol/l) and selumetinib (10 µmol/l). Cell viability was determined using MTT (mean with SEM; n = 3). (B) A549 cells with empty vector (A549 EV) were treated for 72 h with rapamycin (100 nmol/l), torin (250 nmol/l) and selumetinib (10 µmol/l). Colonies were fixed and stained with crystal violet. Three independent experiments were performed and representative pictures were shown.

Supplementary file 6. Table S1. The TMT-based phosphoproteomics analysis was performed on NCI-H23 cells with vector or over-expressing MAL2. The differential phosphorylated proteins including 59 up- and 36 down-regulated proteins are shown.

## Data Availability

All data generated or analyzed during this study are included in this published article and its additional files. The datasets that support the findings of this study are available from the corresponding author on reasonable request.
