# Supplementary figures and images for: T cell differentiation protein 2 facilitates cell proliferation by enhancing mTOR-mediated ribosome biogenesis in non-small cell lung cancer
Source: Discov Oncol. 2022 Apr 18;13:26. doi: 10.1007/s12672-022-00488-z (PMC9016107; doi:10.1007/s12672-022-00488-z)

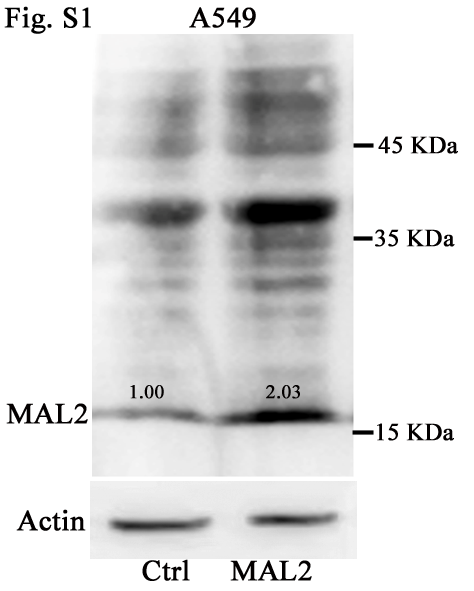

Supplement: Supplementary file 1 — Figure S1. Cell lysates from A549 cells expressing vector control or over-expressing MAL2 were immunoblotted for human MAL2. Molecular weight standards are indicated on the right in kDa. The predicted molecular weight of MAL2 is 19 kDa. The band above 35 kDa was also detected which might be the glycosylated MAL2. A diffuse set of bands were also shown that has been previously described by others. [file 12672_2022_488_MOESM1_ESM.tif]

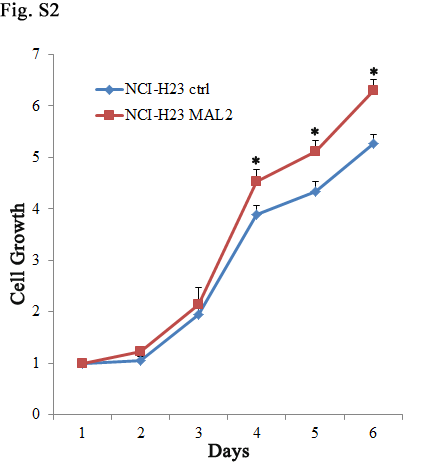

Supplement: Supplementary file 2 — Figure S2. NCI-H23 cells expressing vector control or over-expressing MAL2 were assayed for cell proliferation. Data are mean ± SEM of three replicates from a representative experiment of two independent experiments; t-test, *p < 0.05. [file 12672_2022_488_MOESM2_ESM.tif]

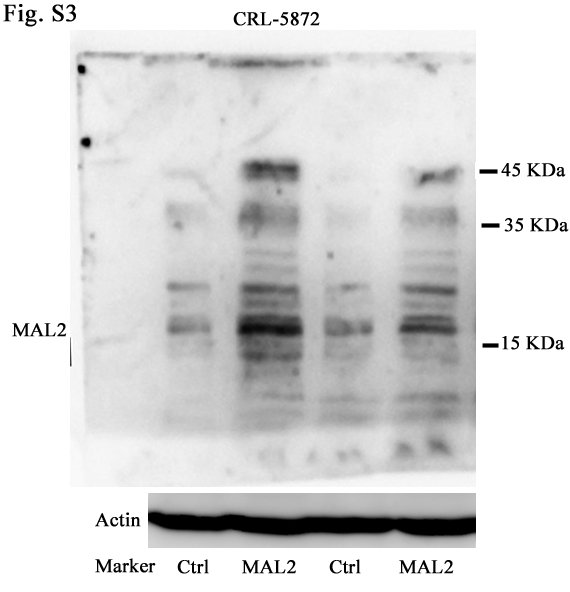

Supplement: Supplementary file 3 — Figure S3. Cell lysates from CRL-5872 cells expressing vector control or over-expressing MAL2 were immunoblotted for human MAL2. The size of 19 kDa MAL2 was shown in Fig. 2C. [file 12672_2022_488_MOESM3_ESM.tif]

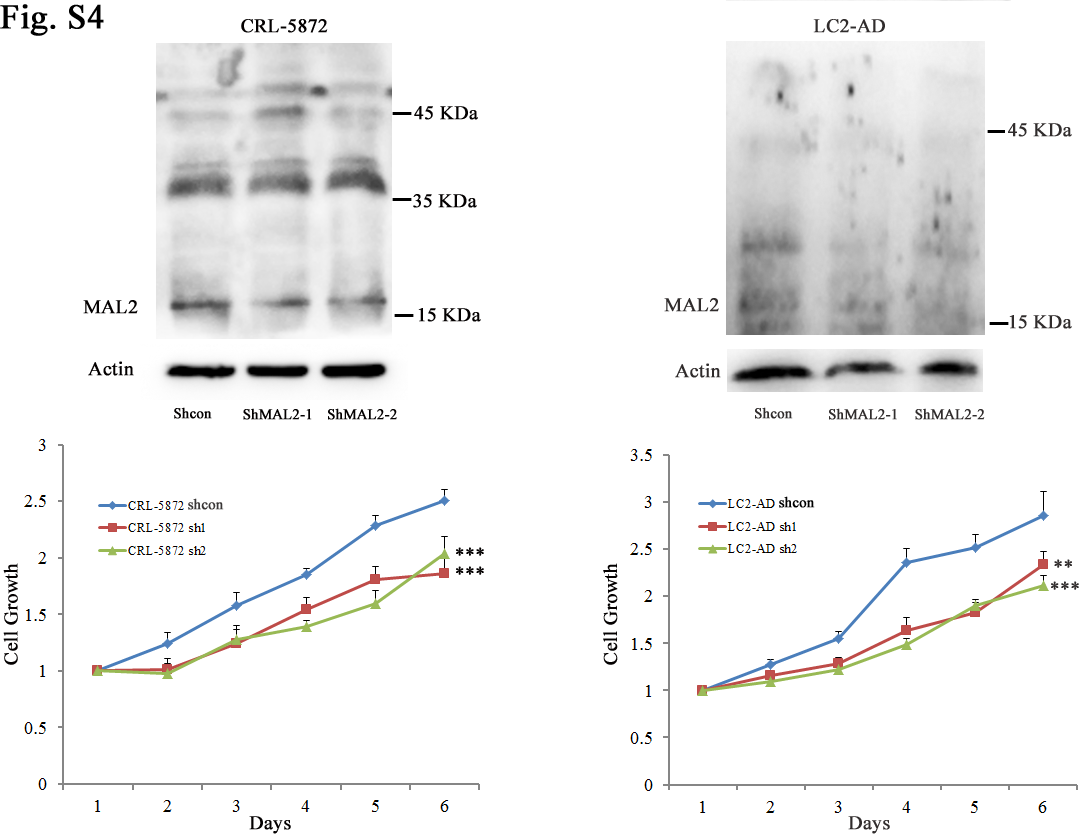

Supplement: Supplementary file 4 — Figure S4. Knockdown effects of MAL2 in CRL-5872 and LC2-AD cell lines. (A, C) Western blotting showed the reduced MAL2 protein levels after 72 h infection of lentivirus with shMAL2 compared with lentivirus with scrambled control shRNA. (B, D) Relative growth curve of CRL-5872 and LC2-AD cells with or without MAL2 knockdown. CRL-5872 cells were seeded in 96-well plates at a density of 3 × 10^3^ per well and LC2-AD cells were seeded in 96-well plates at a density of 4 × 10^3^ per well **P < 0.01, ***P < 0.001. [file 12672_2022_488_MOESM4_ESM.tif]

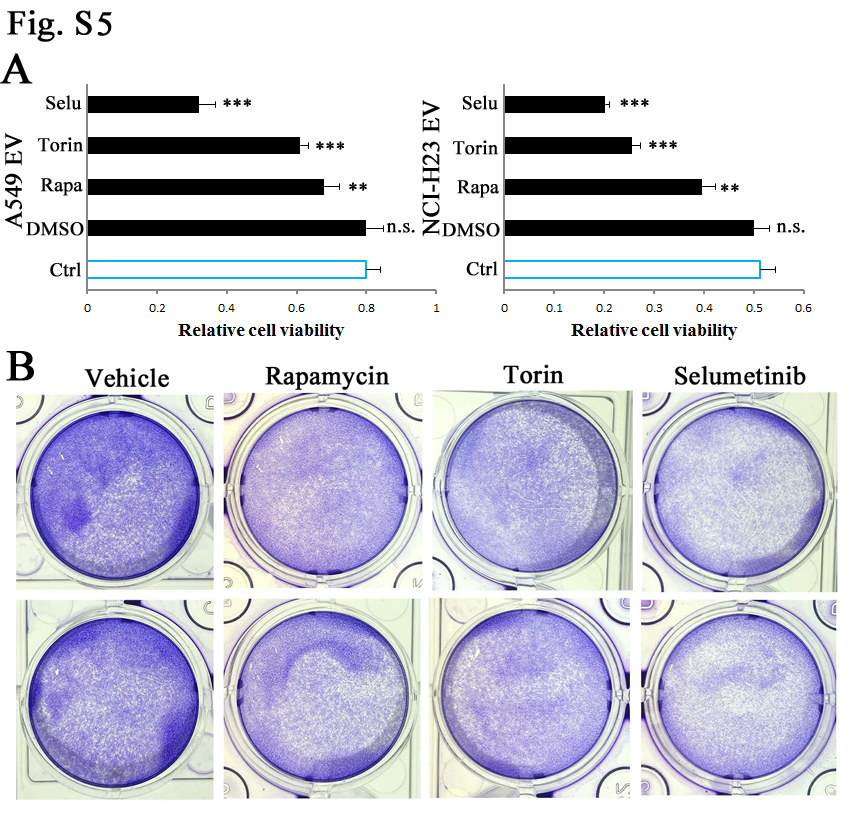

Supplement: Supplementary file 5 — Figure S5. The effects of mTOR- or MEK-specific inhibitors on cell growth. (A) A549 and NCI-H23 with empty vector cells (A549 EV, NCI-H23 EV) were treated for 48 h with rapamycin (100 nmol/l), torin (250 nmol/l) and selumetinib (10 µmol/l). Cell viability was determined using MTT (mean with SEM; n = 3). (B) A549 cells with empty vector (A549 EV) were treated for 72 h with rapamycin (100 nmol/l), torin (250 nmol/l) and selumetinib (10 µmol/l). Colonies were fixed and stained with crystal violet. Three independent experiments were performed and representative pictures were shown. [file 12672_2022_488_MOESM5_ESM.tif]
