# Supplementary material for: T cell differentiation protein 2 facilitates cell proliferation by enhancing mTOR-mediated ribosome biogenesis in non-small cell lung cancer
Source: Discov Oncol. 2022 Apr 18;13:26. doi: 10.1007/s12672-022-00488-z (PMC9016107; doi:10.1007/s12672-022-00488-z)
Supplement: Supplementary file 6 — Table S1. The TMT-based phosphoproteomics analysis was performed on NCI-H23 cells with vector or over-expressing MAL2. The differential phosphorylated proteins including 59 up- and 36 down-regulated proteins are shown. [file 12672_2022_488_MOESM6_ESM.docx]

Table S1: theTMT-based phosphoproteomics analysis was performed on NCI-H23 cells expressing vector control or over-expressing MAL2.

| **Gene name** | **Modifications** | ***P*-value** | **Fold change** | **Number of phospho sites** |
| --- | --- | --- | --- | --- |
| \| RPS6 \| \| --- \| \| PDLIM5 \| \| LMNB1 \| \| DDX21 \| \| PTPN2 \| \| JUNB \| \| CAP1 \| \| NMT1 \| \| MCM4 \| \| GGCX \| \| RRM2 \| \| PAICS \| \| PSMD3 \| \| HECTD1 \| \| DENR \| \| SYNRG \| \| TFDP1 \| \| DDI2 \| \| NOP2 \| \| HJURP \| \| PPP6R3 \| \| TWISTNB \| \| UBTF \| \| TBCB \| \| TWF1 \| \| MYBBP1A \| \| SPDL1 \| \| VIM \| \| SRSF7 \| \| SRPRA \| \| COPA \| \| CAP2 \| \| FAM222B \| \| USP16 \| \| HELLS \| \| SPAG9 \| \| RSL1D1 \| \| KPNA2 \| \| YJU2 \| \| EDC3 \| \| KRT8 \| \| LYN \| \| CAMSAP2 \| \| HDLBP \| \| RPS27 \| \| CNOT3 \| \| BZW2 \| \| POLR3E \| \| WDR70 \| \| ZYX \| \| NCAPG \| \| CLUH \| \| NR2C1 \| \| CD2AP \| \| ESF1 \| \| PARVA \| \| MID1 \| \| LIMA1 \| \| NUP88 \| \| TPD52L2 \| \| TBC1D10B \| \| RNASEK-C17orf49 \| \| ARHGAP35 \| \| TSPYL2 \| \| THOC1 \| \| ZNF185 \| \| KIAA1671 \| \| HDGFL2 \| \| KIAA1191 \| \| TCEA1 \| \| TFDP2 \| \| EXOC1 \| \| PVR \| \| NUCKS1 \| \| CBX1 \| \| PDCD4 \| \| STMN1 \| \| GNAS \| \| FNBP4 \| \| EEF1B2 \| \| ABRAXAS2 \| \| RPL27A \| \| GFPT1 \| \| PAK2 \| \| RTF1 \| \| SLC4A7 \| \| REPS1 \| \| HDGF \| \| GPR39 \| \| RPLP2 \| \| SUB1 \| \| TCEA3 \| \| EEF2 \| \| F11R \| \| AHSG \| | \| Phospho [S236; S240; T241; S242; S244; S246; S247] \| \| --- \| \| Phospho [S228] \| \| Phospho [T19; T20; S23; T25; S391; S393; S404; S405] \| \| Phospho [S13; T15; S71; S89; S121; S164; S168; S171; S173] \| \| Phospho [S304] \| \| Phospho [T255; S259] \| \| Phospho [S34; T307; S308; S310] \| \| Phospho [S47] \| \| Phospho [S31; S32] \| \| Phospho [S11] \| \| Phospho [S20] \| \| Phospho [S27] \| \| Phospho [S6] \| \| Phospho [S631; S632; S640; S1383; S1384] \| \| Phospho [S73] \| \| Phospho [S473; S475] \| \| Phospho [S23] \| \| Phospho [T104; S105; S106] \| \| Phospho [S58; S181; T185; T195; S198; S732; S786] \| \| Phospho [S473] \| \| Phospho [S579; S617] \| \| Phospho [S316; S328] \| \| Phospho [S389; S484] \| \| Phospho [S173] \| \| Phospho [T349] \| \| Phospho [S1159; S1163; T1239; S1241; S1243; T1244; S1248; S1267; T1269] \| \| Phospho [T552; S555] \| \| Phospho [S55; S56; T458; S459] \| \| Phospho [S171; S173; S179; S181; S183] \| \| Phospho [S286; S296; S297; S298; S307] \| \| Phospho [S402] \| \| Phospho [T299; S301] \| \| Phospho [Y136; T138; T143] \| \| Phospho [S415; S423; Y424] \| \| Phospho [S878; S879] \| \| Phospho [S251; S329; T330; S332; S730; S732; S733] \| \| Phospho [T358; S361; S392; S396; S400; T401; T423; S427] \| \| Phospho [T61; S62] \| \| Phospho [S211; S213; S220; S305; S306; S308; S316; S319; S322] \| \| Phospho [S131] \| \| Phospho [S24; S36; S37; S39] \| \| Phospho [S13] \| \| Phospho [S598; S599; S1148] \| \| Phospho [S31; S35; S944] \| \| Phospho [S11; S78] \| \| Phospho [S297; S299] \| \| Phospho [S412; S414] \| \| Phospho [S161; S162] \| \| Phospho [S638] \| \| Phospho [S258; S259; S267; S281; S303; T306; S308; S344] \| \| Phospho [T670; S674; S680; T686; T688; S973; S975; S1015] \| \| Phospho [S664; S669; S670] \| \| Phospho [S64] \| \| Phospho [S232; S233] \| \| Phospho [S153; T311; S312; S313; T319; S327; S657; S663; Y671; S694] \| \| Phospho [S54; S59] \| \| Phospho [S92; S96] \| \| Phospho [S362; S369; S371; S373; S374; S490; S686; S692; S698] \| \| Phospho [S517] \| \| Phospho [S166] \| \| Phospho [T148; T150; S657; S658; S661] \| \| Phospho [S137] \| \| Phospho [S1176; S1179] \| \| Phospho [S16; S17; S18; S20] \| \| Phospho [S560] \| \| Phospho [S152; S153; T154; S155; T158; T447] \| \| Phospho [S1701] \| \| Phospho [S232; S234; S236; S240; S366; S369; S370; S625; S633; S634; S652; S664] \| \| Phospho [T175; S177; S182; S183] \| \| Phospho [T96; S97; S100] \| \| Phospho [S24] \| \| Phospho [S470; S472; S473; S481; S482] \| \| Phospho [S406; S407; S408; T413] \| \| Phospho [Y13; S14; S19; S54; S58; S61; S73; S75; S79; S113; S132; S144; T177; S181; T202; S204; S214; T220; S221; S223; S229; S234; S240] \| \| Phospho [S89; S91] \| \| Phospho [S76; T93; S94; S457] \| \| Phospho [S16; S25; S38] \| \| Phospho [S995] \| \| Phospho [Y113; S116; S124; S427; S429; S431; S432; S464; S963; S964; S965] \| \| Phospho [T87; T88; S90; T93; S95; S106; S112] \| \| Phospho [S368; S372; S375] \| \| Phospho [S68] \| \| Phospho [S261; T262] \| \| Phospho [S141; T169; T178] \| \| Phospho [S53; S58; S60] \| \| Phospho [S84; S233; S407; S1213] \| \| Phospho [S561; S562] \| \| Phospho [S102; S103; S132; S133; S165; T200; S202; S206; T225; S239] \| \| Phospho [S380; T381; T382; S384] \| \| Phospho [S102; S105] \| \| Phospho [S10; S11; S12; S13; S15; S17; S19] \| \| Phospho [S138; S139; S140] \| \| Phospho [T57; T59; T278; S279] \| \| Phospho [S281; S284; S287] \| \| Phospho [S138] \| | \| 7.28E-07 \| \| --- \| \| 2.05E-06 \| \| 2.46E-06 \| \| 4.02E-05 \| \| 0.001392 \| \| 1.36E-05 \| \| 4.18E-05 \| \| 0.002205 \| \| 0.00232 \| \| 1.04E-05 \| \| 2.58E-05 \| \| 3.28E-05 \| \| 2.16E-05 \| \| 7.15E-05 \| \| 0.001141 \| \| 0.001122 \| \| 0.00188 \| \| 0.003904 \| \| 1.98E-05 \| \| 0.006063 \| \| 0.000292 \| \| 0.000257 \| \| 0.009318 \| \| 0.001933 \| \| 0.041498 \| \| 6.82E-05 \| \| 0.007518 \| \| 0.000315 \| \| 0.000291 \| \| 0.000835 \| \| 0.03717 \| \| 0.028635 \| \| 0.000165 \| \| 0.000889 \| \| 0.004786 \| \| 0.000751 \| \| 0.000254 \| \| 6.95E-05 \| \| 0.0001 \| \| 0.006276 \| \| 0.000332 \| \| 0.008091 \| \| 3.06E-05 \| \| 0.000624 \| \| 0.000294 \| \| 0.003496 \| \| 0.009354 \| \| 0.027161 \| \| 4.6E-05 \| \| 0.006328 \| \| 9.18E-05 \| \| 0.000909 \| \| 0.002982 \| \| 0.000224 \| \| 0.000466 \| \| 0.002959 \| \| 0.000563 \| \| 0.000568 \| \| 0.011051 \| \| 0.002816 \| \| 0.025503 \| \| 0.000279 \| \| 0.0177 \| \| 0.023918 \| \| 0.029717 \| \| 0.001156 \| \| 8.36E-05 \| \| 1.32E-05 \| \| 0.006121 \| \| 0.000967 \| \| 0.034995 \| \| 0.00021 \| \| 0.000176 \| \| 0.000702 \| \| 0.002257 \| \| 7E-05 \| \| 0.000199 \| \| 0.005019 \| \| 1.36E-06 \| \| 8.83E-05 \| \| 0.003973 \| \| 0.000999 \| \| 0.00021 \| \| 0.002411 \| \| 0.006002 \| \| 2.23E-06 \| \| 0.000217 \| \| 8.89E-05 \| \| 0.001157 \| \| 0.00023 \| \| 0.000316 \| \| 0.001554 \| \| 0.000178 \| \| 0.001621 \| \| 0.000311 \| | \| 3.27 \| \| --- \| \| 1.97 \| \| 1.85 \| \| 1.57 \| \| 1.44 \| \| 1.43 \| \| 1.42 \| \| 1.40 \| \| 1.39 \| \| 1.38 \| \| 1.38 \| \| 1.38 \| \| 1.38 \| \| 1.37 \| \| 1.35 \| \| 1.33 \| \| 1.33 \| \| 1.32 \| \| 1.32 \| \| 1.32 \| \| 1.31 \| \| 1.31 \| \| 1.31 \| \| 1.30 \| \| 1.29 \| \| 1.29 \| \| 1.28 \| \| 1.28 \| \| 1.27 \| \| 1.27 \| \| 1.26 \| \| 1.26 \| \| 1.26 \| \| 1.25 \| \| 1.25 \| \| 1.25 \| \| 1.25 \| \| 1.24 \| \| 1.24 \| \| 1.24 \| \| 1.24 \| \| 1.24 \| \| 1.24 \| \| 1.24 \| \| 1.23 \| \| 1.23 \| \| 1.23 \| \| 1.23 \| \| 1.22 \| \| 1.21 \| \| 1.21 \| \| 1.21 \| \| 1.21 \| \| 1.21 \| \| 1.21 \| \| 1.21 \| \| 1.20 \| \| 1.20 \| \| 1.20 \| \| 0.83 \| \| 0.83 \| \| 0.83 \| \| 0.82 \| \| 0.82 \| \| 0.82 \| \| 0.82 \| \| 0.82 \| \| 0.81 \| \| 0.81 \| \| 0.80 \| \| 0.80 \| \| 0.80 \| \| 0.79 \| \| 0.79 \| \| 0.79 \| \| 0.78 \| \| 0.77 \| \| 0.77 \| \| 0.76 \| \| 0.76 \| \| 0.75 \| \| 0.74 \| \| 0.73 \| \| 0.73 \| \| 0.72 \| \| 0.71 \| \| 0.69 \| \| 0.69 \| \| 0.68 \| \| 0.64 \| \| 0.61 \| \| 0.60 \| \| 0.57 \| \| 0.54 \| \| 0.45 \| | \| 7 \| \| --- \| \| 1 \| \| 8 \| \| 9 \| \| 1 \| \| 2 \| \| 4 \| \| 1 \| \| 2 \| \| 1 \| \| 1 \| \| 1 \| \| 1 \| \| 5 \| \| 1 \| \| 2 \| \| 1 \| \| 3 \| \| 7 \| \| 1 \| \| 2 \| \| 2 \| \| 2 \| \| 1 \| \| 1 \| \| 9 \| \| 2 \| \| 4 \| \| 5 \| \| 5 \| \| 1 \| \| 2 \| \| 3 \| \| 3 \| \| 2 \| \| 7 \| \| 8 \| \| 2 \| \| 9 \| \| 1 \| \| 4 \| \| 1 \| \| 3 \| \| 3 \| \| 2 \| \| 2 \| \| 2 \| \| 2 \| \| 1 \| \| 8 \| \| 8 \| \| 3 \| \| 1 \| \| 2 \| \| 10 \| \| 2 \| \| 2 \| \| 9 \| \| 1 \| \| 1 \| \| 5 \| \| 1 \| \| 2 \| \| 4 \| \| 1 \| \| 6 \| \| 1 \| \| 12 \| \| 4 \| \| 3 \| \| 1 \| \| 5 \| \| 4 \| \| 23 \| \| 2 \| \| 4 \| \| 3 \| \| 1 \| \| 11 \| \| 7 \| \| 3 \| \| 1 \| \| 2 \| \| 3 \| \| 3 \| \| 4 \| \| 2 \| \| 10 \| \| 4 \| \| 2 \| \| 7 \| \| 3 \| \| 4 \| \| 3 \| \| 1 \| |
| n=95 |  |  |  |  |

Note: the differential phosphorylated proteins including 59 up- and 36 down-regulated proteins in cells with MAL2-overexpression are shown.
